# Tick-borne encephalitis virus in dogs - is this an issue?

**DOI:** 10.1186/1756-3305-4-59

**Published:** 2011-04-13

**Authors:** Martin Pfeffer, Gerhard Dobler

**Affiliations:** 1 Institute of Animal Hygiene & Veterinary Public Health, Centre of Veterinary Public Health, University of Leipzig, An den Tierkliniken 1, 04103 Leipzig, Germany; 2 Bundeswehr Institute of Microbiology, Neuherbergstrasse 11, 80937 Munich, Germany

## Abstract

The last review on Tick-borne encephalitis (TBE) in dogs was published almost ten years ago. Since then, this zoonotic tick-borne arbovirus has been geographically spreading and emerging in many regions in Eurasia and continues to do so. Dogs become readily infected with TBE virus but they are accidental hosts not capable to further spread the virus. They seroconvert upon infection but they seem to be much more resistant to the clinical disease than humans. Apart from their use as sentinels in endemic areas, however, an increasing number of case reports appeared during the last decade thus mirroring the rising public health concerns. Owing to the increased mobility of people travelling to endemic areas with their companion dogs, this consequently leads to problems in recognizing and diagnosing this severe infection in a yet non-endemic area, simply because the veterinarians are not considering TBE. This situation warrants an update on the epidemiology, clinical presentation and possible preventions of TBE in the dog.

## Introduction and epidemiology of TBE

Tick-borne encephalitis (TBE) is the most important tick-borne viral disease of humans in Eurasia with an estimated annual number up to 10,000 cases in Russia and 3,000 cases in Europe [1-5]. TBE is caused by the zoonotic tick-borne encephalitis virus (TBEV), a member of the genus *Flavivirus *within the *Flaviviridae *family [6]. It is classified as a single virus species with three subtypes, i.e. the European subtype, the Siberian subtype (mainly isolates east of the Ural and Siberia) and the Far Eastern subtype (mainly isolates from far-eastern Russia, China and Japan) together representing the geographic distribution of the virus [6,7]. The three TBE virus subtypes are differing with regard to disease severity [1,8,9]. The most severe form of TBE infections with Far Eastern subtype TBE viruses can cause severe febrile illness, frequently associated with encephalitis and a fatality rate up to 35% [10,11]. In contrast, TBE virus infections of the Siberian subtype cause a less severe disease (fatality rate between 1 and 3%). However, these clinical infections have a tendency to become a chronic disease or to cause extremely prolonged infections in some patients [12,13]. Infections caused by European strains typically take a biphasic course. The first viraemic phase presents with fever, malaise, headache, myalgia, sometimes gastrointestinal symptoms, leukocytopenia, thrombocytopenia and elevated liver enzymes after an incubation period of one to two weeks. These non-specific symptoms last for about 2-4 days, often followed by a symptom-free interval of up to one week. The second phase of TBE occurs in approximately one-quarter of the infected patients and shows the clinical signs of meningitis, meningoencephalitis, meningoencephalomyelitis or meningoencephaloradiculitis of different severity. The fatality rate in adult patients is comparable to that caused by the Siberian subtype TBE virus, but is usually less than 2%. However, neurological sequelae may last for months or even years [14].

**Figure 1:**
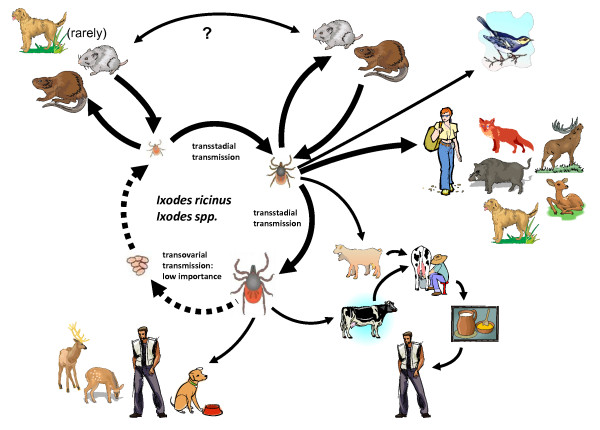
**Schematic drawing of the transmission cycle of tick-borne encephalitis virus**. The dog can serve as host for all three life stages of the Ixodes tick, i.e. the larvae, the nymph and the adult tick. As with humans it is rather the nymphs and even more numerous the adults that feed on dogs. The alimentary infection of humans via TBE virus-contaminated milk is also shown. Although this frequently causes clusters of infection in humans, we are not aware of such an infection route for dogs.

TBE virus is propagated in nature in a transmission cycle consisting of permanently infected tick vectors and wild vertebrate hosts [15]. Within the tick population, the virus is maintained in a transstadial fashion and possibly to a small extent via transovarial transmission to the next developmental stage of the tick's life cycle. Small mammals (mainly rodents), on which larvae, nymphs and adults feed, become infected during the blood meals (Figure 1). Once infected, they serve as virus reservoirs, from which TBE virus is further transmitted in two ways (i) by virus uptake during viremia of the rodent, or (ii) through co-feeding from infected to non-infected ticks feeding on the same host at the same time [16-18]. In addition, TBE virus transmission to humans by raw milk consumption has repeatedly caused clusters of human cases [19-24].

The European TBE virus strains are almost exclusively vectored by *Ixodes ricinus *while *I. persulcatus *serves as vector for the two other subtypes [25,26]. Although the virus has been isolated from several other tick species in nature [27,28], only the two mentioned ixodid tick species appear to play an important role in virus maintenance [17]. *Dermacentor nuttalli*, however, may share this role in southern Siberia and northern Mongolia (unpublished results). Nevertheless, the epidemiology of TBE is tightly linked to the local national history of ixodid ticks [1,4,9,25,29], and the prevalence of infected ticks within the risk areas can vary considerably [1,8,25,29,30]. Countries with high-risk areas (incidence of clinical TBE cases is > 10 per 10^5 ^inhabitants) are Russia, Latvia, Lithuania, Slovenia, and Estonia. TBE is also a significant issue in Germany, the Czech Republic, Poland, Switzerland, Sweden, Finland, Slovakia, and Hungary [31,32]. Although TBE seems to have a minor public health impact in Denmark, France, Greece, Italy and Norway, new TBE foci or possible occurrences of TBE virus were reported here [33-37]. Austria is the only country with progressively decreasing incidences since 1981 due to its vaccination policy [38], but the occurrence of TBE may be relevant to unvaccinated tourists and their accompanying dogs. There are only few studies investigating the TBE prevalence in domestic animals and even less including dogs [39-41].

The last review on Tick-borne encephalitis (TBE) in dogs was published almost ten years ago [42]. In light of the increase of TBE incidence that has been observed in the risk areas in some of the endemic countries mentioned above [4], we feel it warranted to review the current knowledge of TBE in dogs and to plot fields for future research.

## Dogs, ticks and TBE

Tick infestation in dogs is a well recognized world-wide problem. The Ixodidae alone compromise 702 tick species in 14 genera [43], but not all of them parasitize dogs. Depending on the geographic area, the tick species associated with dogs differ considerably. In North America, for example, virtually all ticks found on dogs are brown dog ticks (*Rhipicephalus sanguineus*), American dog ticks (*Dermacentor variabilis*), Rocky Mountain wood ticks (*D. andersoni*), Western dog ticks (*D. occidentalis*), Lone Star ticks (*Amblyomma americanum*), Gulf Coast ticks (*A. maculatum*), deer ticks (*Ixodes dammini*), blacklegged ticks (*I. scapularis*), and woodchuck ticks (*I. cookei*) [44,45]. Studies from northern Brazil further demonstrate that the tick species composition varies between urban dogs (exclusively *R. sanguineus *infested) and dogs from rural areas (at least four species including *R. sanguineus*) [46,47]. As outlined above, none of the previously listed tick species is involved in the natural transmission of TBE virus, because TBE virus is not yet prevalent in these regions. However, *I. cookei *and *I. scapularis *were shown to be naturally infected with the closely related Powassan virus in North America [48,49]. Experimental data on transmission potential and vector capacity of ticks exclusively occurring in the New World are not available for TBE virus. The most abundant ticks in northern Europe, the castor bean tick, *I. ricinus*, and the Taiga tick, *I. persulcatus*, are transmitting TBE virus. Both of them parasitize dogs. In Japan, *I. ovatus*, another Ixodes tick species frequently feeding on dogs, is supposed to vector TBE virus [50]. With their furry coats, the close proximity to the ground and their behavior in seeking and exploring, dogs are 50 to 100 times more likely to come in contact with disease-carrying ticks than humans [51]. Walking the dog and leisure activity has been identified as important risk factors for humans to aquire a TBE virus infection [25,52]. Being a dog owner in an endemic area is thus enhancing the risk to be bitten by a tick, simply because of the time spent outdoors and the increased exposure when compared with people not regularly walking a dog. In addition, ticks attached to the dogs are brought home where they may infest humans, in particular children, who are also playing with the animals indoors. Meanwhile TBE has become an accepted issue in human travel medicine [53,54]. Since the establishment of the EU, traveling between European (and to some extent to Eurasian) countries has become much easier and millions of holiday or business tourists from non-endemic countries are visiting endemic areas within the time of the year when ticks are active and transmission of TBE virus can occur. While an increasing but still small percentage of these people consider to get vaccinated against TBE, none is aware of the fact that the accompanying dogs are much more likely to become infected due to the reasons outlined above [54].

## Clinical signs and Diagnosis

Based on the few case reports available (see Table), TBE manifests with almost the identical symptoms as seen in clinical human cases. However, the clinical cases seen in dogs, which are later confirmed in the laboratory as TBE, present as very severe and have almost exclusively a fatal outcome. Incubation period is supposed to equal the one known for humans and thus is supposed to be between one and two weeks. However, because clinical TBE is rarely diagnosed, this period is an estimate made in analogy from the human situation, but since even experimental infections of puppies usually do not result in clinical illness [55], the incubation period in dogs may be shorter than one or longer than two weeks. Detailed descriptions of the clinical development have been published [56-60]. Common in the clinical course of TBE in dogs is the elevated body temperature (up to 41.4°C) and change in behavior (denying food, increased aggressiveness, skittishness or apathia). All ill dogs showed motor failures either on the forhand or the rear legs with retarded proprioception and hyporeflexy in front and/or rear legs. More detailed clinical inspection with neurological examination reveals paresis, mostly tetraparesis, to generalized ataxia and tetraplegia, myoclonus, vestibular syndrome (Strabismus), sensibility loss in the head area but cervical hyperalgesia, facial nerve paralysis, anisocoria, nystagmus, miosis or loss of eye lid closing reflex (see Table 1). These signs reflect the multifocal neurological disorder in the cerebrum and the brain stem. A single case description links TBE as possible cause of optic neuritis in a Siberian Husky [61].

**Table 1:** Tick-borne encephalitis virus in Carnivora and Canidae

Year	Animal species ^a^	Clinincal symptoms	Viremia? ^b^	Reference- location	Ab-response? ^c^
Experimental infections					

1946	Wolf puppies i.c.	Paresis, convulsions, encephalitis, death	Virus isolation	[70]	High Ab-titers
1956	Adult foxes i.c.	No clinic	n.d.	[71]	n.d.
1958	Fox puppies	Fever	viremia	[72]	High Ab-titers
1959	Dog i.n.	encephalitis	n.d.	[73]	n.d.
1969	Foxes, badgers, weasels via *Ixodes ricinus*	Meningencephalitis (foxes)	yes	[74]	n.d.
1972	Dog puppies s.c. and via *Haemaphysalis inermis *and *Dermacentor marginatus *ticks	No clinic in puppies infected via tick, weakness in extremities in s.c. infected puppies	Low level viremia found irregularly	[55]	Yes

Natural infections					

1960	n.d.	Encephalitis	n.d.	[75] Sweden	Yes (first documented clinical case of TBE in a dog)
1970	1 Landseer (4.5 years old)	Aggressiveness, fever, tremor paresis, meningitis, seizures, cramp of front legs	Yes (first isolate form a dog after natural infection)	[56,76] Switzerland	n.d.
1993	5 dogs (2 Rottweiler, 1 Greyhound, 1 Husky, 1 Golden Retriever)	Ataxia, tetraparesis, fever, seizures of grand male-type	n.d.	[60] Switzerland	IgM in CSF in 2 dogs. All were euthanized and diagnoses were confirmed by IHC
1994-1997	3 Husky, 1 Terrier-mix, 1 Rottweiler, 1 Irish setter, 1 Bastard, 1 Pekingese	Convulsion, tremor, ataxia, hyperesthesia, hemi-/tetraplegia, recumbency, opisthotonus, seizures, anisocoria, miosis, nystagmus	n.d.	[63] Austria	Immunohistology in brain tissue positive in 5 dogs, but pathohistological changes were similar in the remaining three dogs
1998	1 Rottweiler (4 years old), 1 Newfoundland dog (6 years old)	Fever, hyperaesthesia, seizures opistotonus, facialparesis, strabismus, sensoric loss (head)	n.d.	[58] Germany	Yes (both dogs)
2001	1 (Riesenschnauzer, 2.5 years old)	Fever, aggressiveness, ataxia, shivering	n.d.	[77] Sweden	Yes
2002	1 dog	Fever, ataxia, shivering, agressivness, quadriplegia	n.d.	[77] Sweden	Yes
2006	1 dog	Ataxia, tremor, sensoric loss	n.d.	[78] Sweden	Yes
2007	2 dogs	Fever, ataxia, tremor, pain, head shaking. Both fully recovered after 1 year	n.d.	[79] Sweden	Yes
2009	1 mix-breed (12 years old)	Polypneu, ataxia, weakness, diffuse pain (euthanized)	n.d.	[80] Italy	n.d. (PCR and IHC of brain tissue positive)

Serosurveillance studies					

1988-1991	255	No clinic	n.d.	[81] Sweden	18 seropositive
1993-1998	About 1.000 dogs	No clinic relating to CNS symptoms	n.d.	[64] Germany	Between < 2% (northern states) and 31% (Bodensee area)
1994 & 1995	10 sentinel dogs each year (*Ixodes ovatus*)	No clinic	3 virus isolates, Far-Eastern subtype	[50] Japan	Japan, high Ab-titers upon seroconversion
1997-1998	151 dogs	In three Rottweiler dogs with meningoencephalitis or encephalitis	n.d.	[59] Czech Republic	5 seropositive (3.3%)
1999	552 dogs	Clinical signs in 57 of the seropositive dogs	n.d.	[66] Austria	133 seropositive (24.1%, ELISA); 110 confirmed by NT (19.9%)
1998-2003	317 dogs	Not observed	n.d.	[82] Southern Norway	52 seropositive (16.4%)
2002	54 healthy & 56 dogs with neurological symptoms	Neurological symptoms not further specified	n.d.	[83] Germany	17/54 seropositive; 30/56 seropositive
2005-2006	125 dogs	Not observed	n.d.	[39] Denmark	30% ELISA-, 4.8% NT-antibodies
2009	960 dogs	Not observed	n.d.	[84] Belgium ^d^	1 seropositive (0.1%) ^d^

a = infection routes: i.c., intra cranial; i.n., = intra nasal; s.c.; = sub cutaneous

b = n.d., not determined

c = Ab, antibody; ELISA = enzyme-linked immunosorbent assay; NT = neutralization test; IHC = immunohistochemistry

d = Belgium is still considered to be non-endemic for TBE. A travel history of the seropositive dog to endemic areas in France and Germany may explain this result.

Since clinical signs vary, laboratory confirmation of the etiological agent causing the encephalitic outcome is needed. Haematology may show physiological leucocyte counts, but the differential blood count may indicate a monocytosis and a lymphopenia or a leucopenia with physiological counts of both monocytes and lymphocytes [58]. Elevated total leucocyte and mononuclear cell counts as well as high protein concentration in the liquor are typical indicators for an encephalitis and are commonly described in cases of clinical TBE in the dog [58,59].

Specific diagnostic procedures to confirm clinically suspected TBE include detection of TBE virus in the serum during viremia by one of the many published RT-PCRs or real-time RT-PCRs [e.g. 62]. Viral antigen can also be detected by immunohistopathology of brain tissues after necropsy [60,63]. In most cases, the initial supposition does not include TBE and thus this diagnostic will probably be requested too late after the infection to detect virus. Hence, laboratory confirmation of TBE is mostly done by serology. Antibody titers against TBE virus can be measured either by indirect immunofluorescence assays (IFA, Euroimmune, Lübeck, Germany) or an all-species ELISA (Progen Biotechnik GmbH, Heidelberg, Germany). Detection of IgM or the four-fold rise in IgG antibodies in a serum pair taken about two weeks apart confirms the diagnosis. Serology in flaviviral infections in humans is notoriously hampered by cross-reactivity between other infections or vaccinations such as dengue-, Japanese encephalitis- or yellow fever viruses. In the case of dogs, the only possible cross-reactivity which may interfere with the serological confirmation of a TBE case could be an infection of the dog with West Nile virus (WNV), another flavivirus that has a growing geographic range in Eurasia and which is partially overlapping with the known distribution of TBE. However, dogs do not become readily infected with WNV, so at least to what is known to date, serological diagnosis of TBE in dogs is confirmative.

Another issue to be considered in serodiagnosing TBE is that only a rise in the specific antibody titres in paired sera is conclusive when seroprevalence rates of more than 30% are known in endemic areas [64]. Although not particularly shown for dogs in endemic areas, it is known from cattle in Hungary that animals of older age groups have significantly higher seroprevalence rates than those of younger age groups. This could either argue for longevity of TBE virus-specific antibodies or a higher frequency of encountering TBE virus-infected ticks over time [40]. However, whether this applies to dogs as well and thus has to be kept in mind while verifying the diagnosis serologically is not known and might be subject of further studies. Nevertheless, there is no case definition formulated for TBE in dogs, but positive serological results in conjunction with time spent in known endemic areas is what we consider a confirmed case. The known history of tick bites is a further epidemiological link, but not necessary for confirming the diagnosis. This is mainly because dogs are supervised and inspected with different intensities and thus ticks may easily be overlooked.

## Pathology, Treatment and Prevention

The dominating pathological picture is that of a massive encephalitis while visceral organs are without gross lesions or histopathological findings. The meningoencephalitis is non-suppurative and characterized by necrosis of both neurons and glia cells. Almost the entire brain shows typical signs of inflammation like perivascular cuffs and infiltration (lymphocytes, histiocytes and plasma cells), neuronophagy, glial nodules or diffuse gliosis [60,63]. Pathological changes were most prominent in the brain stem and the cerebellum [65]. Based on the probably most comprehensive investigation by Kirtz in 1999 [66], the neurostructures related to the clinical symptoms are the thalamus (66%, fever and 54% altered consciousness and behavior), the cerebral cortex (54%, the same symptoms as above), the mesencephalon (42%, proprioceptive deficit), the spinal cord (37%, motor neuron deficit), meninges (21%, hyperalgesia in the neck), brainstem (20%, head tilt, facial paresis, nystagmus, strabismus), cerebral cortex and thalamus (12%, seizures). Taken together, the neuropathology of TBE in dogs is largely consistent with the neuropathology of TBE in humans [63].

Because no causal treatment exists to fight TBE, treatment is solely symptomatic. Emphasis has to be put on preventing secondary harm to the patient itself as well as the owner during convulsions and aggressive behavior. For that reason, therapy should include resting as well as anticonvulsive and sedative medication. The application of dexamethasone and its possible beneficial effect is controversially discussed: given too early in the course of infection, the glucocorticoide may prolong the destructive viral activity, but when administered during the reconvalence phase, a fast decline of symptoms was observed [60,66]. Non-steroidal anti-inflammatory drugs (NSAID) are best used to combat the high fever, and antibiotics should be given to prevent secondary bacterial infections, in particular pneumonia [57]. Most of the few dogs, that survived a clinical TBE needed between a half and one year to fully recover. As with in human cases, an intensive physical therapy seems to be a key issue in the process of training and compensating the loss of neuronal damage caused by the severe inflammation of the central nervous system.

Preventative measures are therefore very important and they are primarily targeting the prevention of tick bites. One problem when dealing with three-host ticks (Figure 1) is the fact that the majority of reproducing ticks is not feeding on the dog but rather on wildlife hosts. As we are limited in our ability to manage ticks on their "natural" hosts, reinfestation of dogs is common requiring protracted use of acaricides [67]. There are many powerful anti-ectoparasite drugs with acaricide function of up to four weeks on the market, which can be easily applied in a spot-on or pour-on by the dog owner. Since a dog needs to be walked, this is the most effective way to prevent infection via ticks in an endemic area. Numerous studies have shown the often excellent efficacy of products containing acaricides such as amitraz, fipronil, and permethrin against ticks infesting dogs [reviewed in [67]]. If a dog is treated with an effective acaricide, and encounters only a few ticks, it is likely that all these ticks will be killed. However, if tick exposure is considerably larger, a few ticks may still be remaining on these dogs and, when observed by pet owners, they may perceive a lack of efficacy. One of the most commonly practiced solutions to this problem is to increase the frequency of acaricide application on the dog [67]. Since the current classes of acaricides, which are orders of magnitude less toxic for the dog and the environment than formerly used DDT or lindane formulations, this practice does not cause harm to the dog patient. Topical application also reduces systemic levels of the active ingredients and is localized directly on the skin, the critical interface for bloodsucking ticks [68]. The costs and benefits of regularly using topicals to reduce tick infestation needs to comprise the estimated and potential burden of ill health due to TBE virus infection and other tick-borne diseases or owner attitudes towards ectoparasitism as well as the estimated burden due to the documented attributable toxic effects of the preventative compound [68]. Although it still seems that clinical TBE in dogs is a rare event, such an analysis is likely to conclude that the benefits of topicals outweigh their slight risk of toxicity.

In Europe, there are currently two vaccines licensed for human use, including special formulations or vaccination schemes for infants and older patients. None of the two companies (Novartis and Baxter) has their TBE vaccines licensed for any animal use including dogs. However, vaccines from both companies have been successfully used to vaccinate various animal species (e.g. sheep, goat, roe deer, dogs) without any adverse effect. Antibody titers measured either in IFA or ELISA suggest, that in all cases, a protective immune response was induced through vaccination with human vaccines (69, M. Niedrig, RKI, Berlin, pers. communication, own unpublished results). Hence, vaccination of dogs should be considered an effective preventive measure in highly endemic areas.

## Conclusion

Seroprevalence studies in areas of Eurasia where TBE virus is endemic clearly show that dogs are highly susceptible to an infection. For yet unknown reasons, clinical manifestation seems to be a rare event, but then likely to have a fatal outcome. Increasing numbers of case reports describing clinical TBE in dogs are found in the recent literature with some coming from previously non-endemic areas. This raises concerns with regard to a further geographical expansion of TBE virus-endemic areas, an increasing prevalence of TBE virus in the vector ticks and/or a change in virulence of the TBE virus strains involved. Regardless of the underlying reasons, these observations have implications for travel medicine in dogs. One major aim of this article is to raise awareness of the clinical picture of TBE in dogs so that this diagnosis may be considered in cases of neurological disorders even in yet non-endemic areas, but for example in dogs with a respective travel history. The currently known TBE virus-endemic areas should be known, so travel recommendations can be made including the application of the topical acaricides. Vaccines for human have impressively demonstrated that the incidence in humans can be dramatically reduced in highly endemic areas with the right vaccination policy. First results indicate that these vaccines can be used in dogs, but comprehensive studies on the safety and efficacy of the existing human vaccines in dogs are warranted in order to have alternative prevention measures at hand when they are needed. With increasing numbers of TBE cases in humans, a likewise increase of such cases in dogs may occur in the near future. Hence, we should monitor TBE virus infections in dogs regardless whether they present with clinical signs or only as a serconversion without an overt clinic.

## Competing interests

The authors declare that they have no competing interests.

## Authors' contributions

Both authors contributed equally to this work and have approved the final version of the manuscript.
